# Discovery of Therapeutics Targeting Oxidative Stress in Autosomal Recessive Cerebellar Ataxia: A Systematic Review

**DOI:** 10.3390/ph15060764

**Published:** 2022-06-19

**Authors:** Sze Yuen Lew, Michael Weng Lok Phang, Pit Shan Chong, Jaydeep Roy, Chi Him Poon, Wing Shan Yu, Lee Wei Lim, Kah Hui Wong

**Affiliations:** 1 Department of Anatomy, Faculty of Medicine, Universiti Malaya, Kuala Lumpur 50603, Malaysia; szeyuenlew@gmail.com (S.Y.L.); michaelphang4572@gmail.com (M.W.L.P.); 2 Neuromodulation Laboratory, School of Biomedical Sciences, Li Ka Shing Faculty of Medicine, The University of Hong Kong, 21 Sassoon Road, Pokfulam, Hong Kong, China; u3005073@connect.hku.hk (P.S.C.); jaydeep@connect.hku.hk (J.R.); chpoonac@connect.hku.hk (C.H.P.); yuwsw@connect.hku.hk (W.S.Y.)

**Keywords:** autosomal recessive cerebellar ataxia, rare neurodegenerative disease, genetic mutation, preclinical model, oxidative stress, antioxidant pathway and therapy

## Abstract

Autosomal recessive cerebellar ataxias (ARCAs) are a heterogeneous group of rare neurodegenerative inherited disorders. The resulting motor incoordination and progressive functional disabilities lead to reduced lifespan. There is currently no cure for ARCAs, likely attributed to the lack of understanding of the multifaceted roles of antioxidant defense and the underlying mechanisms. This systematic review aims to evaluate the extant literature on the current developments of therapeutic strategies that target oxidative stress for the management of ARCAs. We searched PubMed, Web of Science, and Science Direct Scopus for relevant peer-reviewed articles published from 1 January 2016 onwards. A total of 28 preclinical studies fulfilled the eligibility criteria for inclusion in this systematic review. We first evaluated the altered cellular processes, abnormal signaling cascades, and disrupted protein quality control underlying the pathogenesis of ARCA. We then examined the current potential therapeutic strategies for ARCAs, including aromatic, organic and pharmacological compounds, gene therapy, natural products, and nanotechnology, as well as their associated antioxidant pathways and modes of action. We then discussed their potential as antioxidant therapeutics for ARCAs, with the long-term view toward their possible translation to clinical practice. In conclusion, our current understanding is that these antioxidant therapies show promise in improving or halting the progression of ARCAs. Tailoring the therapies to specific disease stages could greatly facilitate the management of ARCAs.

## 1. Introduction

Autosomal recessive cerebellar ataxias (ARCAs) are a heterogeneous group of rare, complex, progressive and disabling inherited neurodegenerative disorders [1]. Typically, ARCAs are early-onset (before the age of 30) diseases involving the cerebellum, brainstem and spinocerebellar tracts [2]. The worldwide prevalence of ARCAs was estimated to be three to five per 100,000, in which ARCAs represent about half of all hereditary ataxias [3,4].

Notably, ARCAs should be suspected particularly in individuals with multiple affected siblings in a single generation and/or consanguinity in parents, whereas sporadic cases can occur before the age of 40, depending on the carrier frequency in the population of origin [5,6]. ARCAs manifest diverse features associated with their complex phenotypes including peripheral neuropathy, pyramidal and extrapyramidal involvement, oculomotor abnormalities, mental retardation, cognitive impairment, seizures, retinopathy and hypogonadism [7]. Although most of the ARCAs have unique features that can be distinguished clinically and pathologically, the overlapping of phenotypes often complicates the diagnosis [8]. Advancement in gene screening techniques such as next-generation sequencing (NGS) have led to the identification of more than 90 genes associated with ARCAs [9,10]. The most common ARCAs are Friedreich’s ataxia (FRDA), ataxia-telangiectasia (A-T) and ataxia oculomotor apraxia type 1 (AOA1) and type 2 (AOA2) [11].

Oxidative stress has been suggested to be implicated in the pathogenesis of numerous neurodegenerative diseases, including hereditary ataxias [12]. Many studies have been conducted to validate the therapeutic roles of antioxidants in ARCAs [13,14,15,16,17]. However, their findings showed that antioxidants have only marginal therapeutic benefits for the management of ARCAs. These antioxidants were found to be ineffective or only partially effective on the symptoms of ARCAs, which could be due to lack of understanding of their modes of action targeting oxidative stress. In addition, the focus is often on the clinical outcomes and not the underlying molecular mechanisms [16]. To date, no systematic review has focused on the therapeutic roles of antioxidants and their associated modes of action for the management of ARCAs.

This systematic review aims to examine and synthesize the current research on therapeutic strategies targeting oxidative stress in the treatment of ARCAs published from 1 January 2016 onwards. We also critically examine various cellular and animal models that have been developed to study the role of oxidative stress and free radicals in ARCAs. Understanding the molecular triggers and regulation of signaling networks is critical for developing novel therapies for ARCAs. These preclinical findings may pave the way for clinical studies of antioxidant therapies in patients with ARCAs.

## 2. Involvement of Oxidative Stress and Mitochondrial Dysfunction in the Pathogenesis of ARCAs

### 2.1. Ataxia-Telangiectasia (A-T)

Ataxia-telangiectasia (A-T), also known as Louis-Bar syndrome, is a progressive multisystem disease that may become apparent during infancy or early childhood. A-T is characterized by gait and truncal ataxia, slurred speech, conjunctival telangiectasias, polyneuropathy, hyperkinesia, hyporeflexia, choreoathetosis, frequent infections and hypersensitivity to ionizing radiation with increased susceptibility to cancer [18,19]. It is the second most common form of ARCA in childhood after Friedreich’s ataxia (FRDA) [20]. The worldwide prevalence of A-T was estimated to be between 1 per 40,000 and 1 per 300,000 [21].

**Figure 1:**
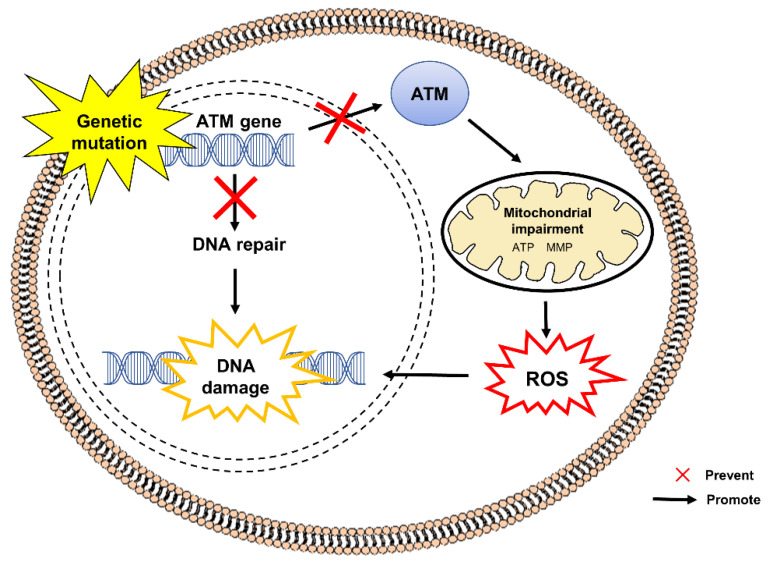
Mutation in the ATM gene in the pathogenesis of A-T. The defective ATM gene results in the disruption of DNA repair mechanisms that are critical for maintaining the integrity of genomic DNA and subsequent accumulation of unregulated DNA damage. An absence or deficiency of ATM protein contributes to the impairment of mitochondria, leading to excessive production of ROS. Dysregulated ROS signaling further accelerates DNA damage. ATM, ataxia-telangiectasia mutated; ATP, adenosine triphosphate; DNA, deoxyribonucleic acid; MMP, mitochondrial membrane potential; ROS, reactive oxygen species.

Figure 1 shows the pathogenesis of A-T caused by mutations in the ataxia-telangiectasia mutated (ATM) gene (11q22.3–23.1). Null mutation of the gene contributes to the under-expression of functional ATM protein, a member of phosphoinositide 3-kinase (PI3K) family [22,23]. ATM is known for its role in ensuring cell survival by regulating DNA double-strand break (DSB). Recent studies have suggested that DNA-DSB is not the sole contributor to A-T as it has also been found that disruption of the equilibrium between reactive oxygen species (ROS) production and antioxidant defense is involved in the pathogenesis of A-T. ATM acts as a sensor for oxidative stress and maintains redox homeostasis [23]. ATM deficiency results in hypersensitivity against ROS [24,25], eventually triggering DNA-DSB and oxidative stress-induced DNA damage [26]. Ambrose et al. [27] revealed that A-T cells possess aberrant changes in mitochondrial dynamics with reduced mitochondrial membrane potential (MMP) as well as increased expression of mitochondrial DNA repair and ROS-scavenging genes, namely the polymerase gamma (POLG), mitochondrial-targeted topoisomerase 1 (TOP1mt), peroxiredoxin 3 (Prx3) and superoxide dismutase 2 (SOD2), denoting activation of multiple signaling pathways in response to oxidative stress and compensatory mechanisms against mitochondrial dysfunction in A-T cells.

### 2.2. Ataxia with Oculomotor Apraxia (AOA)

Ataxia with oculomotor apraxia type 1 (AOA1) is characterized by childhood onset of slowly progressive cerebellar ataxia followed by peripheral neuropathy and quadriplegia with loss of ambulation around 7 to 10 years after the onset. Patients with AOA1 often experience ataxia and/or chorea as the early predominant symptoms. As the disease progresses, they exhibit symptoms including oculomotor apraxia, peripheral neuropathy, areflexia, strabismus, dystonia, hypoalbuminemia and hypercholesterolemia [28]. Cases of AOA1 have been reported in Tunisia [29], Germany [30], Italy [31], the United States [32], northern Europe [33,34] and France [35]. AOA1 is the most common form of ARCA in the Japanese population. In Portugal, AOA is the second most common form of ARCA after FRDA [16] with an overall prevalence estimated to be 0.41 per 100,000 in which 3.6% of ARCA cases are AOA1 [36].

AOA1 results from mutation in the aprataxin (APTX) gene encoding aprataxin protein. Aprataxin is crucial in mitochondrial DNA repair and resolving abortive DNA ligation intermediates. Aprataxin deficiency impairs mitochondrial morphology and functional network, which are crucial for the maintenance of mitochondrial DNA integrity and interchanging mitochondrial material [34,37]. APTX-deficient cells undergo mitochondrial stress with altered expression of a mitochondrial inner membrane fusion protein, namely optic atrophy type 1 (OPA1) and the components of oxidative phosphorylation complexes, including complexes I, II, III and IV [38]. Impaired autophagic removal of damaged mitochondria causes excessive production of mitochondrial ROS.

Ataxia with oculomotor apraxia type 2 (AOA2) is characterized by onset of ataxia between age 10 and 25 years, sensorimotor neuropathy, postural tremor, dystonia, strabismus, oculomotor apraxia, cerebellar atrophy and elevated serum level of alpha-fetoprotein (AFP) [39]. In addition, Le Ber et al. [40] revealed dyadic pursuit and gaze-evoked nystagmus in patients with AOA2. Cases of AOA2 have been reported in North Africa [41,42], Japan [43], Middle East [44], Germany [45] and North America [46]. The worldwide prevalence was estimated to be 1 per 900,000 [18,35].

AOA2 results from a mutation in the senataxin (SETX) gene that encodes senataxin protein, a nuclear protein involved in DNA damage response [47]. AOA2 cells are highly sensitive to oxidative DNA damage induced by hydrogen peroxide (H_2_O_2_), camptothecin and mitomycin C. Another novel form of AOA has been reported to exhibit increased sensitivity against exogenous and endogenous agents causing single-stranded DNA breaks followed by increased levels of poly (ADP-ribose)polymerase (PARP-1) auto-poly (ADP-ribosyl)ation, ROS and reactive nitrogen species (RNS) [48].

**Figure 2:**
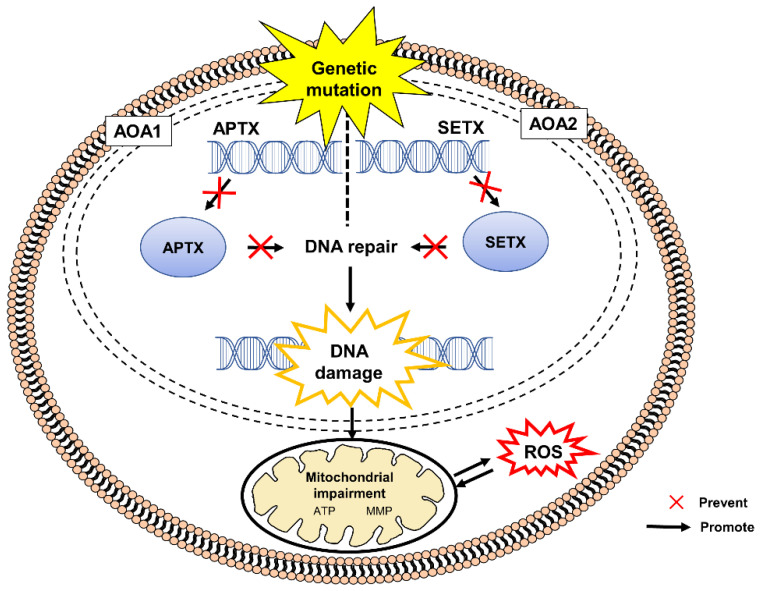
Mutations in the APTX and SETX genes in pathogenesis of AOA1 and AOA2, respectively. The defective genes result in the disruption of DNA repair mechanisms that are critical for maintaining the integrity of genomic DNA and subsequent accumulation of unregulated DNA damage. An absence or deficiency of APTX or SETX protein contributes to the impairment of mitochondria leading to excessive production of ROS. AOA, ataxia with oculomotor apraxia, APTX, aprataxin, ATP, adenosine triphosphate; SETX, senataxin, DNA, deoxyribonucleic acid, MMP, mitochondrial membrane potential; ROS, reactive oxygen species.

Figure 2 shows the pathogenesis of AOA1 and AO2 caused by mutations in APTX and SETX genes, respectively.

### 2.3. Ataxia with Vitamin E Deficiency (AVED)

Ataxia with vitamin E deficiency (AVED) is characterized by progressive ataxia with isolated vitamin E deficiency, and an age of onset between age 4 and 20 years. The phenotypes of AVED are very similar to those of FRDA but cardiomyopathy and glucose intolerance are less frequent [5]. Patients with AVED often exhibit symptoms such as loss of vibration and joint position sense, dysdiadochokinesia, positive Romberg and Babinski signs, tendon areflexia, head titubation, decreased visual acuity, dystonia, scoliosis and pes cavus [2,5]. Although most of the cases originated in the Mediterranean region, AVED had been reported in Japan [49], Padua [50], Alsace region of France [35], Tunisia [51] and southeast Norway [52]. The worldwide prevalence of AVED was estimated to be less than 1 per 1,000,000.

**Figure 3:**
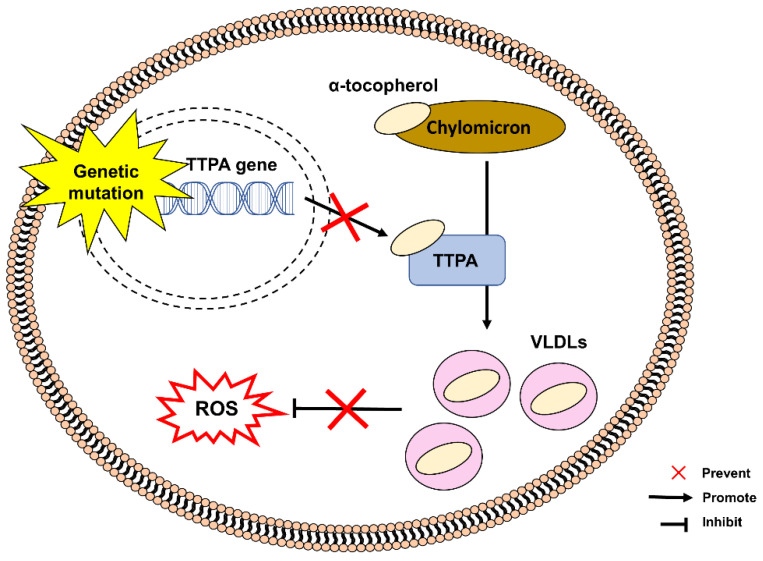
Mutation in the TTPA gene in the pathogenesis of AVED. An absence or deficiency of TTPA protein prevents the transfer of α-tocopherol from chylomicrons to very-low-density lipoproteins (VLDLs). Insufficient circulation of α-tocopherol contributes to vitamin E deficiency, which can increase the susceptibility to oxidative stress. TTPA, α-tocopherol transfer protein, VLDLs, very-low-density lipoproteins, ROS, reactive oxygen species.

Figure 3 shows the pathogenesis of AVED caused by mutations in a gene encoding α-tocopherol transfer protein (TTPA), responsible for transferring α-tocopherol from chylomicrons to very-low-density lipoproteins (VLDLs). TTPA deficiency causes low circulating α-tocopherol concentrations, leading to vitamin E deficiency and inadequate protection against oxidative stress [53,54]. TTPA is a critical regulator of body status of vitamin E by facilitating the secretion of α-tocopherol from hepatocytes and enabling the absorption and transportation of vitamin E between membranes of intracellular organelles. TTPA is primarily expressed in hepatocytes, cerebellum and placenta [54,55,56].

### 2.4. Autosomal Recessive Spastic Ataxia of Charlevoix-Saguenay (ARSACS)

Autosomal recessive spastic ataxia of Charlevoix-Saguenay (ARSACS) is characterized by early-onset ataxia between age 1 and 14 years, spasticity, axonal and demyelinating neuropathy, retinal hypermyelination, pes cavus and hammer toes [5,57]. Originating from the regions of Charlevoix and Saguenay-Lac-St-Jean (SLSJ), Quebec, ARSACS is more common in French-Canadians than in any other population. Cases of ARSACS have been reported in Tunisia [58,59], Turkey [60], Japan [61], Italy [62], Spain [63], Netherlands [64], Belgium [65,66,67], India [68], Australia [69], the United States, Brazil and North Africa [18,34]. The exact worldwide prevalence of ARSACS is unknown [70].

**Figure 4:**
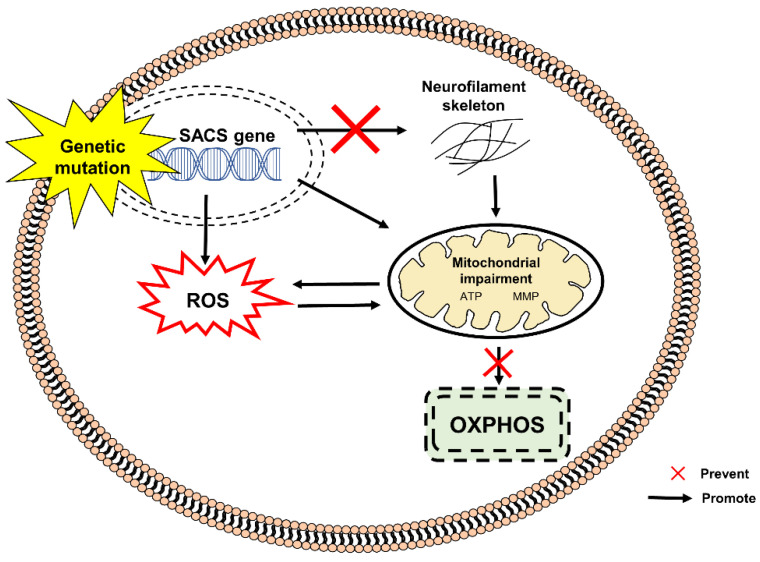
Mutation in the SACS gene in the pathogenesis of ARSACS. An absence or deficiency of SACS protein contributes to the excessive production of ROS, modification of neurofilaments and mitochondrial impairment. These events lead to further generation of ROS and disruption of OXPHOS. ATP, adenosine triphosphate; MMP, mitochondrial membrane potential; SACS, sacsin; OXPHOS, oxidative phosphorylation; ROS, reactive oxygen species.

Figure 4 shows the pathogenesis of ARSACS caused by bi-allelic mutations in SACS gene encoding sacsin, a multidomain protein of 4579 amino acids. Sacsin acts as a chaperone in mediating protein folding/unfolding via the ubiquitin-proteasome signaling pathway, as well as regulating mitochondrial dynamic and assembly of neurofilaments [34,66,71]. Sacsin deficiency has been demonstrated to alter the neurofilament network and mitochondrial function in animal models and fibroblasts derived from patients with ARSACS. Disruption of mitochondrial dynamics, particularly mitochondrial fission, affects mitochondrial transport from the soma to distal axonal and dendritic regions causing neuronal death [72,73]. Increased oxidative stress and mitochondrial dysfunction in fibroblasts derived from patients with ARSACS lead to reduced basal respiration rate, adenosine triphosphate (ATP)-linked respiration rate, maximal mitochondrial respiration rate, respiratory chain activities and mitochondrial ATP synthesis [74].

### 2.5. Friedreich’s Ataxia (FRDA)

Friedreich’s ataxia (FRDA) is characterized by early-onset ataxia around puberty, with loss of coordination and cardiomyopathy. Patients with FRDA usually experience motor symptoms including gait ataxia, weakness in their lower extremities, peripheral neuropathy, dysphagia and dysarthria; as well as non-motor symptoms, comprising skeletal deformities, diabetes and hypertrophic cardiomyopathy. Cases of FRDA have been reported in Europe, the Middle East, North Africa and India, with a worldwide prevalence estimated to be at 1 to 2 per 50,000 [1,75]. In Europe, the prevalence of FRDA varies between 1 per 20,000 and 1 per 725,000. The highest prevalence was reported in southern France, northern Spain and Ireland, whereas the lowest prevalence was reported in Scandinavia and Russia [76].

**Figure 5:**
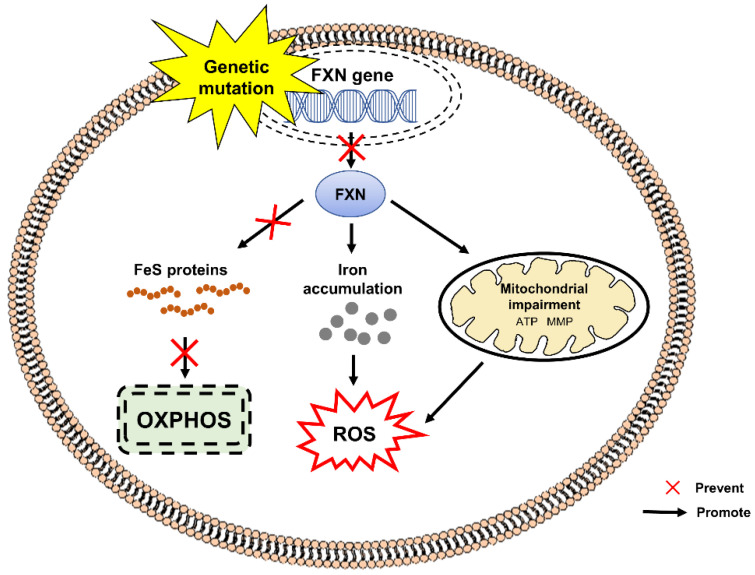
Mutation in the FXN gene in the pathogenesis of FRDA. An absence or deficiency of frataxin protein contributes to the disruption of FeS clusters biogenesis, disrupting oxidative OXPHOS. In addition, the protein deficiency also contributes to abnormal accumulation of iron and mitochondrial impairment, leading to excessive production of ROS. ATP, adenosine triphosphate; FXN, frataxin; FeS, iron-sulfur; MMP, mitochondrial membrane potential; OXPHOS, oxidative phosphorylation; ROS, reactive oxygen species.

Figure 5 shows the pathogenesis of FRDA caused by mutation in frataxin (FXN), the gene encoding frataxin protein. Expansion of guanine-adenine-adenine (GAA) trinucleotide in the first intron of the FXN gene [77] results in decreased expression of mitochondrial protein frataxin, which is responsible for cellular iron metabolism and iron-sulfur (FeS) clusters biogenesis in the mitochondrial matrix [78,79]. Frataxin deficiency causes increased production of intracellular ROS and impaired formation of FeS clusters such as heme, electron transport chain (ETC) complexes I-III and aconitase [80]. Excessive iron accumulation in the mitochondrial matrix, mitochondrial dysfunction, mitochondrial energy imbalance with decreased ATP production and increased sensitivity to oxidative stress are the key features of FRDA [81]. Several studies have shown increased levels of biomarkers of oxidative stress in the urine and blood samples from patients with FRDA [82,83,84,85].

## 3. Standard Therapeutic Options for ARCAs and Their Adverse Effects

The AVED is, in fact, potentially treatable. Van de Warrenburg et al. [86] reported that AVED may be treated with oral administration of high-dose vitamin E of up to 800–1500 mg/day, which is usually given over a patient’s lifetime. Although recovery may be moderate and incomplete, oral supplementation of vitamin E has been shown to stabilize and improve neurological symptoms in patients with AVED [34,87]. Moreover, early therapy with vitamin E has been demonstrated to reverse ataxia and prevent disease manifestation in pre-symptomatic patients [51]. Besides, oral supplementation of baclofen is effective in controlling spasticity, preventing tendon shortening and joint contractures in patients with ARSACS in the early stage of the disease [16,88].

On the other hand, no therapies have been shown effective to date for A-T, AOA1 and AOA2. Nevertheless, it is possible to reduce the neurological symptoms of A-T by dopamine agonist, anticholinergics and selective serotonin reuptake inhibitors (SSRI) [89]. Similarly, FDA has not approved or authorized treatments any for FRDA. Idebenone treatment at early stages of FRDA aims to reduce the progression of cerebellar manifestations and to improve mitochondrial function. Idebenone is a synthetic benzoquinone (analog of coenzyme Q_10_, CoQ_10_) with potent antioxidant activity that promotes ATP production by regulating mitochondrial ETC [90,91]. Canadians had access to idebenone following its approval in 2008 until its withdrawal from the Canadian market by the end of April 2013 due to the failure of clinical studies to confirm the claims of its effectiveness [15]. Although idebenone is generally safe and well tolerated, the findings from clinical studies have been inconclusive. It failed to show improvements in any neurological ataxia indices and cardiomyopathy [92,93,94].

## 4. Involvement of Antioxidant Defense Mechanisms for the Management of Autosomal Recessive Cerebellar Ataxia

The redox equilibrium is essential in regulating cell homeostasis. Enzymatic antioxidants, namely superoxide dismutase (SOD), catalase (CAT), glutathione peroxidases (GPx) and non-enzymatic antioxidants, namely glutathione (GSH), vitamin A, C, E, lipoic acid and CoQ10 mitigate any form of oxidative/nitrosative stress or its consequences [95,96].

Antioxidants, either endogenously generated or externally supplied, are capable of scavenging ROS and reducing oxidative stress in ARCAs, which could open a new direction for translational ARCA research. Compounds that increase the expression of antioxidant genes such as Omaveloxolone (Omav), which can pharmacologically activate antioxidative transcription factor NRF2 (nuclear factor erythroid 2–related factor 2) could be a viable strategy to mitigate primary or secondary oxidative damage in ARCAs. Research could also prioritize the molecular pathways that are shared across several ARCAs [97,98,99]. The NRF2 is a master regulator of redox homeostasis, responsible for directly or indirectly modulating the expression of key antioxidant enzymes in response to oxidative stress [100,101]. Within the nucleus, NRF2 interacts with small musculoaponeurotic fibrosarcoma (MAF) proteins to form heterodimers, which are then bound to antioxidant response element (ARE) to promote downstream gene expression of antioxidant enzymes [101,102].

Moreover, the involvement of mitochondria in the pathogenesis of AOA, ARSACS and FRDA could be targeted for pharmacological interventions. Therefore, the development of mitochondria-targeted antioxidants that selectively block mitochondrial oxidative damage and prevent apoptosis, particularly ferroptosis, is highly warranted for the management of ARCAs. Ferroptosis is a type of regulated cell death of necrotic nature characterized by iron accumulation, lipid peroxidation and release of damage-associated molecular patterns (DAMPs).

## 5. Materials and Methods

### 5.1. Search Strategy

We performed a literature search of PubMed, Web of Science, and Science Direct Scopus for relevant peer-reviewed articles published from 1 January 2016 onwards. The following search terms were used: (“antioxidant defense*” OR “antioxidant therapy*”) AND (“autosomal recessive cerebellar ataxia*” OR “autosomal recessive hereditary ataxia*”) AND (“alternative medicine*” OR “complementary medicine*” OR “compound*” OR “drug*” OR “herb*” OR “natural product*” OR “peptide*” OR “plant*” OR “protein*” OR “proteomic*” OR “traditional Chinese medicine*”).

### 5.2. Eligibility Criteria

Studies were considered eligible if they met the following inclusion criteria: (i) pre-clinical (in vitro and in vivo) studies, (ii) study model for ARCAs as the primary disorder, and (ii) articles published in English. Studies were not included according to the exclusion criteria: (i) studies considering ataxia as a secondary symptom, (ii) studies targeting non-motor symptoms, (iii) studies using chemical substances to induce ataxia, (iv) review articles, (v) meta-analysis, (vi) conference abstracts or proceedings, and (vii) articles written in languages other than English.

### 5.3. Data Extraction and Analysis

After removing duplicates, titles and abstracts were screened based on the inclusion criteria. Disagreements on the eligibility of the study or on the extraction of data were resolved through discussions between team members. The findings were extracted independently and narrated to the best of our ability, taking into account the inconsistencies in the methodology or experimental designs of the retrieved studies.

## 6. Results

### Study Selection

**Figure 6:**
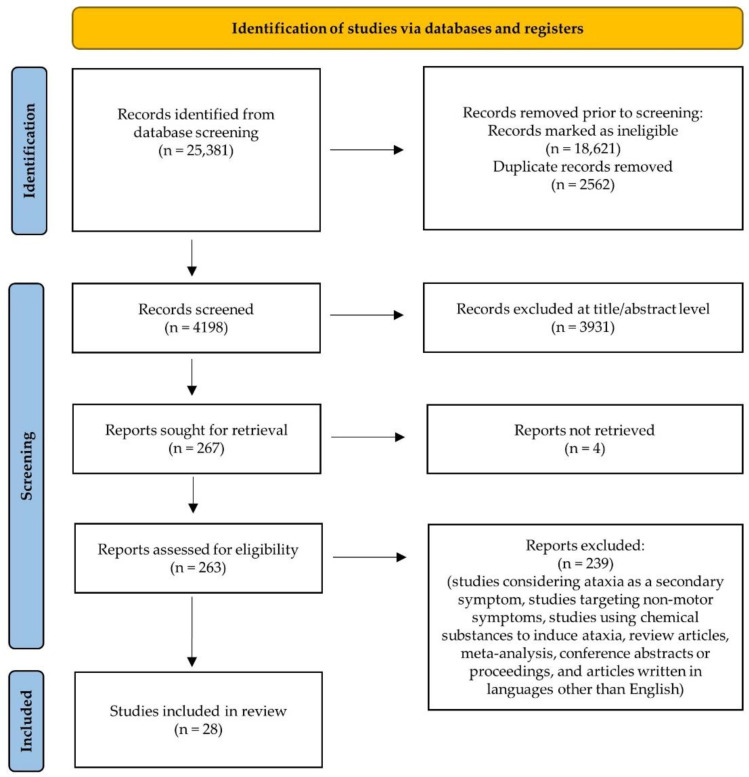
PRISMA flow chart for the identification of relevant studies.

The literature search yielded 25,381 publications from PubMed, Web of Science, and Science Direct Scopus. After removing duplicate studies, 4198 studies remained and were further screened by titles and abstracts. Overall, 263 full-text articles were retrieved for further assessment and evaluation, of which 239 were excluded according to the eligibility criteria. A total of 28 studies were included in this review. Figure 6 shows the PRISMA flow chart for the identification of relevant studies.

## 7. Discussion

### 7.1. Antioxidant Defense Mechanisms and Antioxidant Therapies in Ataxia-Telangiectasia

The findings and modes of action of the current antioxidant therapies for the management of ataxia-telangiectasis (A-T) are summarized in Table 1.

#### 7.1.1. α-Lipoic Acid

Alpha-lipoic acid (α-LA), also known as thioctic acid, is an organosulfur compound derived from caprylic acid used for the treatment of metabolic syndromes by activating the antioxidant defense system [106,107,108,109]. The protective effects of 10 to 20 µM α-LA were evaluated on fibroblasts derived from patients with A-T following exposure to IL-1β, an inflammatory cytokine. IL-1β promoted nuclear factor kappa-light-chain-enhancer of activated B cells (NF-kB) activation and mRNA expression and protein level of IL-8, a pro-inflammatory cytokine and a reliable serum biomarker in patients with A-T and enhanced intracellular and mitochondrial ROS production, causing mitochondrial dysfunction. Administration of α-LA has been shown to inhibit NF-kB activation, mRNA expression and protein level of IL-8, and reduce ROS production, therefore restoring mitochondrial function by preventing MMP dissipation and increasing ATP production [26].

#### 7.1.2. CRISPR/Cas9

The clustered regularly interspaced palindromic repeats (CRISPR)/Cas9 is a gene-editing system that allows permanent modification of a genomic target sequence [110]. Ovchinnikov et al. [103] applied the system to induced pluripotent stem cells (iPSCs) derived from patients with A-T by introducing corrected ATM genes into TTAA piggyBac excision sites. Gene correction of one allele in iPSCs was able to restore the protein level of ATM. Exposure of gene-corrected iPSCs to gamma radiation demonstrated phosphorylation of ATM downstream targets involved in DSB repair, namely serine-1981 and serine-824, indicating the restoration of DNA damage response and cell cycle control. Additionally, the gene-corrected iPSCs showed restoration of ATM kinase function following DNA-DSBs induced by etoposide, indicated by an increase in KRAB-associated protein 1 (KAP1) level and a decrease in γ-H2A.X, mitochondrial ROS, cleaved caspase 3 and cell death following 250 μM H_2_O_2_-induced oxidative stress and 20 mM or 100 mM 2-deoxy-D-glucose (2DG)-induced metabolic stress [103]. The findings suggest that correction of ATM gene and restoration of the protein level of ATM can be beneficial in protecting A-T cells against DNA-DSBs and oxidative stress-induced cell death.

#### 7.1.3. Dexamethasone

Dexamethasone (DEX) is a synthetic glucocorticoid commonly used as an antiemetic in the treatment of nausea and vomiting following chemotherapy [111]. Although DEX improved neurological symptoms and motor deficits in children with A-T, its precise mechanisms have not been elucidated [112].

The antioxidant properties of DEX were evaluated in lymphoblasts derived from patients with A-T [104]. Administration of 100 nM DEX attenuated ROS level by increasing the levels of reduced GSH and total GSH, indicating an enrichment of GSH pool and endogenous antioxidant capacity. Furthermore, quantitative PCR also revealed that mRNA expression of genes involved in GSH synthesis, namely the glutamyl-cysteine ligase catalytic subunit (GCLC), glutamyl-cysteine ligase modifier subunit (GCLM), glutathione synthetase (GSS) and glutathione reductase (GSR) was also upregulated. Further investigation showed that the ability of DEX to enhance the antioxidant defense system was mediated by NRF2 activation through nuclear translocation of NRF2, which was further confirmed by an increase in NADPH quinone oxidoreductase 1 (NQO1), the downstream target of NRF2 [104]. In addition, Biagiotti et al. [22] showed that 100 nM DEX suppressed mRNA expression and protein level of Kelch-like ECH-associated protein 1 (KEAP1), an inhibitor of NRF2, and promoted nuclear translocation of NRF2 in lymphoblasts derived from patients with A-T.

#### 7.1.4. Genetic Suppressor Element 4

Genetic suppressor elements (GSEs) are cDNA fragments encoding peptides or RNA antisense inhibitors. The GSE 4 is derived from GSE 24.2 peptide, a fragment of dyskerin TRUB domain [113,114]. Iarricio et al. [114] reported the cytoprotective and antioxidant properties of GSE4 in dyskerin-mutated epithelial cells. The GSE4 peptide was incorporated into a transfer vector followed by delivery of the transgene into fibroblasts and lymphoblasts derived from patients with A-T [105]. The peptide suppressed oxidative stress-induced DNA damage in A-T cells by increasing the activity of 8-oxoguanine DNA glycosylase 1 (OGG1) and decreasing the level of 8-oxoguanine (8-oxoG) and accumulation of oxidative DNA base damage, specifically at telomeres and mitochondrial DNA. The OGG1 enzyme is able to recognize and excise 8-hydroxydeoxyguanosine (8-OHdG), a product of oxidatively damaged DNA formed by hydroxyl radical, singlet oxygen and direct photodynamic action. A high level of mitochondrial 8-OHdG has been reported to be correlated with increased mutations, deletions and loss of mitochondrial DNA, and apoptosis. Thus, the generation of low levels of ROS was also detected in parallel with enhanced mRNA expression of SOD1, reduced mRNA expression of interleukin-6 (IL-6) and inhibition of pro-apoptotic pathway mediated by activated p38 [105]. However, expression of the peptide failed to repair DNA-DSB caused by the lack of ATM activity.

Moreover, A-T is also considered a secondary telomerase disease due to diminished telomere length along with downregulated mRNA expression of telomerase reverse transcriptase (TERT) [115,116]. The peptides were able to increase mRNA expression of TERT, contributing to reconstitution of telomerase activity and elongation. Further investigation showed that these protective effects were associated with the suppression of oxidative stress in the telomere and mitochondria [105].

**Figure 7:**
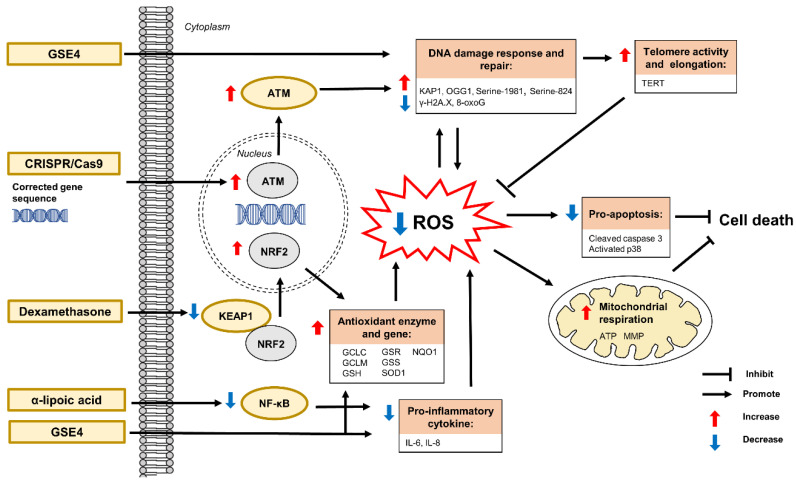
Antioxidant therapies against A-T. The defective ATM gene leads to the disruption of DNA damage response and repair, excessive production of ROS, and impairment of mitochondria. Antioxidant therapies can modulate ATM, NRF2, TERT, serine-1981, serine-824, KAP1 and γ-H2A.X expressions, and mitochondrial impairment, resulting in the restoration of DNA damage response and repair, antioxidant enzyme and gene levels, mitochondrial function, and telomere activity and elongation. This also leads to the attenuation of ROS production and pro-apoptotic activities. ATM, Ataxia-telangiectasia mutated; ATP, adenosine triphosphate; DNA, deoxyribonucleic acid; GCLC, glutamyl-cysteine ligase catalytic subunit; GCLM, glutamyl-cysteine ligase modifier subunit; GSEs, genetic suppressor elements; GSH, glutathione; GSR, glutathione reductase; GSS, glutathione synthetase; IL, interleukin; KAP1, KRAB-associated protein 1; KEAP1, Kelch-like ECH-associated protein 1; MMP, mitochondrial membrane potential; NQO1, NAD(P)H quinone oxidoreductase 1; NF-kB, nuclear factor kappa-light-chain-enhancer of activated B cells; NRF2, nuclear factor erythroid 2–related factor 2; OGG1, 8-oxoguanine DNA glycosylase-1; ROS, reactive oxygen species; SOD1, superoxide dismutase 1; TERT, telomerase reverse transcriptase; γ-H2A.X, gamma-H2A histone family member X; 8-oxoG, 8-oxoguanine.

Figure 7 shows a schematic of all the modes of action of current antioxidant therapies for the management of A-T.

### 7.2. Antioxidant Defense Mechanisms and Antioxidant Therapies in Friedreich’s Ataxia

The findings and mode of action of the current antioxidant therapies for the management of FRDA are summarized in Table 2.

#### 7.2.1. α-Tocotrienol Quinone

Alpha-tocotrienol quinone (EPI-743) is an analog of α-tocopheryl quinone (EPI-A0001) and structurally related to vitamin E. It can be broadly classified as an antioxidant developed for the potential treatment of mitochondrial diseases [140]. EPI-743 demonstrated clinical improvement in neurological function and is well-tolerated in patients with FRDA [141]. Also, 1 µM EPI-743 has been found to increase mRNA expression and protein levels of NRF2 and its downstream genes, namely NQO1, heme-oxygenase-1 (HO-1) and γ-glutamyl cysteine ligase (GCL) regulated by of KEAP1-DJ-1-p62 in fibroblasts derived from patients with FRDA [117]. GCL is a rate-limiting enzyme that catalyzes the biosynthesis of GSH [142]. Activation of NRF2 increased mRNA expression and the protein level of frataxin. However, GSH has been observed to remain completely stable despite an increase in GCL expression, raising the possibility that increased GCL expression may not always translate to elevated GSH level [117], In addition, administration of 1 µM EPI-743 decreased lipid peroxidation and rescued the morphological defects of the mitochondria, suggesting restoration of redox homeostasis modulated by ferroptosis-related genes, namely the FXN, NRF2, SOD2, glutathione peroxidase 4 (GPX4), and GCL in fibroblasts derived from patients with FRDA [118].

On the other hand, administration of 1 µM EPI-743 demonstrated similar effects in neural stem cells (NSCs) isolated from the embryonic cortex of FXN knock-in/knock-out (KIKO) mice with an increase in mRNA expression and protein levels of NRF2 and its downstream targets, (NQO1 and HO-1). Activation of NRF2 attenuated ROS level and prevented early phenotypic defects in neurogenesis through the recovery of neuronal morphology and differentiation [119].

#### 7.2.2. Diazoxide

Diazoxide is a benzothiadiazine derivative and a potent vasodilator used for the treatment of systemic hypertension, capable of lowering pulmonary vascular resistance and causing longstanding improvement of symptoms in patients with primary pulmonary hypertension [143]. It targets potassium-sensitive ATP channels of the inner mitochondrial membrane. The primary roles of these channels include regulation of mitochondrial respiration and the alteration of MMP. Additionally, the channels modulate mitochondrial matrix volume and ROS synthesis for neuronal survival [144,145,146].

Santoro et al. [120] evaluated the protective effects of diazoxide in lymphoblasts derived from patients with FRDA and YG8sR FRDA mice. Administration of 100 μM diazoxide appeared to elevate the mRNA expression and protein level of frataxin and mTOR-S6K-signaling pathway and promoted nuclear translocation of NRF2 in the lymphoblasts. These findings suggest that the protective effects of diazoxide are associated with activation of mTOR and its downstream targets, including NRF2. Accordingly, ramamycin, an mTOR kinase inhibitor, repressed the protein level of frataxin. A growing body of evidence suggests that mTOR regulates homeostasis by influencing protein synthesis, transcription, autophagy, metabolism, and organelle biogenesis and maintenance. Following the promising protective effects demonstrated in an in vitro model, diazoxide was further investigated in a YG8sR FRDA mouse model. Oral administration of 3 mg/kg diazoxide improved fine motor coordination and balance assessed by beam walking assay and stride length assessed by footprint analysis. Furthermore, diazoxide upregulated mRNA expression and protein level of frataxin and NRF2 in the cerebellum and heart, which leads to increased aconitase activity and reduced protein oxidation in the brain, liver and pancreas [120].

#### 7.2.3. Dimethyl Fumarate

Dimethyl fumarate (DMF) is a methyl ester synthesized through the esterification of fumaric acid. Recently, DMF has been shown to exert neuroprotective and immunomodulatory effects associated with endogenous antioxidant mechanisms and mitigation of oxidative stress in in vivo models [147,148].

Petrillo et al. [117] evaluated the antioxidant properties of DMF in fibroblasts derived from patients with FRDA. Administration of 30 µM DMF has been shown to upregulate mRNA expression of NQO1, HO-1 and GCL mediated by NRF2 and KEAP1-DJ-1-p62, resulting in the increased production of GSH. Furthermore, the mRNA expression of FXN is comparable to that of asymptomatic carriers.

Jasoliya et al. [121] provided insight into pathophysiological mechanisms underlying FXN-dependent deficiency in perturbing mitochondrial bioenergetics. FXN deficiency decreased mitochondrial copy number in fibroblasts and whole blood obtained from patients with FRDA, and FXN KIKO mouse model. The NRF-1 plays a significant role in coordinating the transcriptional regulation of all nuclear-encoded mitochondrial subunits, which controls the mitochondrial transcription factor A (mTFA) protein, the key regulator of mtDNA transcription and replication [149]. Mitochondrial biogenesis could estimate some downstream consequences of frataxin deficiency such as disease severity, progression and therapeutic effectiveness.

In line with these observations, administration of 10 and 30 µM DMF has been shown to upregulate the mRNA expression and protein level of frataxin in fibroblasts derived from patients with FRDA, leading to mitochondrial biogenesis via an elevation in mitochondrial copy number. Additionally, administration of 3–10 mg/kg DMF increased mRNA expression and protein level of frataxin in lymphoblasts derived from patients with FRDA, spleen of YG8 mice and cerebellum of FXN KIKO mice, suggesting that increased transcription initiation and elongation arises from the removal of R-loops and transcriptional silencing of the FXN locus [122]. The YG8R mouse and cellular models exhibit GAA repeat-mediated FXN gene silencing associated with increased DNA methylation, and reduced levels of aconitase activity and mRNA expression of PGC-1α and SOD2.

#### 7.2.4. Elamipretide

Elamipretide (SS-31) is a Szeto-Schiller (SS) tetrapeptide targeting cardiolipin (CL), a phospholipid localized in the inner mitochondrial membrane associated with the stabilization of mitochondrial cristae structure and improving mitochondrial bioenergetics [150,151,152]. Zhao et al. [123] evaluated the antioxidant properties of SS-31 on fibroblasts and lymphoblasts derived from patients with FRDA. Administration of 50 nM SS-31 has been found to increase the protein level of frataxin, ATP and NAD+/NADH ratio, and restore MMP and morphology of mitochondria following frataxin deficiency. Enzymatic activities of FeS, including mitochondrial aconitase and complex II and III were upregulated due to increased frataxin. In addition, decreased ROS production was observed due to increased protein levels and activities of SOD and CAT, denoting enhanced ability of the cells to counteract frataxin deficiency-induced oxidative stress.

Peripheral sensory neurons are particularly susceptible to neurodegeneration FRDA, resulting in the loss of proprioception. Liu et al. [124] explored the potential of SS-31 in Y47 and YG8R FRDA mice models and their isolated dorsal root ganglia (DRG). The primary outcome was the upregulation of mRNA expression of frataxin. Intraperitoneal injection of 1 mg/kg SS-31 has been found to improve motor function, accompanied by a decreased number of hypertrophic cardiomyocytes and abnormal Purkinje cells, reduced cytoplasmic vacuolization in DRG and lesions in the dentate nuclei, and restored damaged myelin in the spinal cord.

#### 7.2.5. Exenatide

Exenatide is a glucagon-like peptide 1 (GLP1) analog derived from exendin-4. It is a mimetic of incretin that has been used as an adjunctive therapeutic for type 2 diabetes mellitus [153,154]. Igoillo-Esteve et al. [125] investigated the antioxidant properties of exenatide on iPSC-derived β cells and DRG sensory neurons obtained from patients with FRDA and FXN KIKO mice. The iPSC-derived sensory neurons can recapitulate native DRG sensory neurons and also display voltage-gated channels and express proteins characteristic of nociception, mechanoreception, and proprioception.

Administration of 50 or 500 nM exenatide has been revealed to restore the protein level of frataxin, FeS apoprotein (aconitase, NADH: ubiquinone oxidoreductase core subunit s3, NDUFS3), lipoic acid-containing proteins [2-oxoglutarate dehydrogenase E1 component (OGDH) and pyruvate dehydrogenase (PDH)] in iPSCs sensory neurons [125]. Correspondingly, increased frataxin leads to increased ATP production, basal respiration and maximal respiratory capacity, resulting in the restoration of mitochondrial function. Further, subcutaneous implantation of 10 μg exenatide in the interscapular area has been demonstrated to improve glucose tolerance, β cell function and insulin secretion in the islets of Langerhans and confer neuroprotective effects against DRG sensory neurons in the cerebellum and cerebrum of FXN KIKO mice via increasing the protein levels of frataxin and ferrochelatase [125].

#### 7.2.6. Gold Cluster Superstructure

Gold quantum clusters are metal nanoclusters synthesized via the formation of hydrogen bonds between the capping ligands of gold cores. Previous investigations have demonstrated the ability of gold quantum clusters to attenuate ROS level in 3T3 fibroblasts [155,156].

The gold cluster superstructures identified as Au8-pXs have been regarded as an improved derivative of gold quantum clusters, demonstrating cytoprotective properties against mesenchymal stem cells (MSCs) derived from bone marrow samples of patients with FRDA [126]. A catalytic activity of 5 to 10 µM Au8-pXs attenuated mitochondrial ROS production and increased the protein level of frataxin, resulting in restoration of mitochondrial function and bioenergetic capacity, ATP level, ETC function and MMP dissipation. Modulation of autophagic flux involving LC3-I, LC3-II, p62 and autophagy-related 7 (ATG7), frataxin-related proteins (NRF2 and cysteine desulfurase [NFS1]), and dynamin-related proteins controlling mitochondrial fusion, mitofusin 1 (MFN1) and fission (dynamin-related protein 1, DRP1) contributed to the protective effects of Au8-pXs.

In addition, systemic injection of 10 µM Au8-pXs in the YG8sR mice has been shown to improve motor deficits and neuromuscular function assessed by rotarod and footprint tests, and treadmill and forelimb grip tests, respectively, and cardiac contractility. Villa et al. [126] observed that matrix metalloproteinase (MMP9) deficiency reduced collagen deposition in the skeletal muscle and cardiac fibrosis, concomitant with a decrease in tumor necrosis receptor-associated factor 6 (TRAF6) and collagen synthesis, regulating myocardial necroptosis and remodeling.

Taken together, the protective effects were attributed to reduced ROS production, and therefore resulted in decreased 4-hydroxynonenal (4-HNE), an aldehyde product of phospholipid peroxidation and 8-oxo-2′-deoxyguanosine (8-oxodG), reduced LC3-II/LC3-1 ratio and upregulated N-ethylmaleimide-sensitive fusion protein (NSF) in the cerebellum associated with an increase in PGC-1α and a decrease in PPARγ, regulation of antioxidant enzymes, namely peroxiredoxin 2 (PRDX2) and glutathione S-transferase mu 1 (GSTM1), and activation of NRF2 in the DRG, denoting a reversion of autophagic flux impairment and restoration of ARE. Consequently, increased levels of ATP and ETC promote mitochondrial function [126].

#### 7.2.7. *Hericium erinaceus*

The culinary-medicinal mushroom *Hericium erinaceus*, also known as the lion’s mane mushroom, is well known for its diverse therapeutic activities related to neuroprotection [127,157,158,159,160] and neuroregeneration [161,162,163] attributed to its antioxidant properties [127,160,164]. In a study by Lew et al. [127], a standardized aqueous extract of *H. erinaceus* has been shown to possess higher total phenolic content and more potent reducing power compared to solvent extracts. The ability of exogenous antioxidants to scavenge free radicals has been suggested to contribute to the protective effects against BSO-induced oxidative stress in FRDA fibroblasts. FRDA fibroblasts were challenged under conditions in which GCL had been blocked pharmacologically with BSO, and therefore mimicked the actual pathogenesis in FRDA. Preliminary findings show that *Hericium erinaceus* restored the depleted GSH/oxidized glutathione (GSSG) ratio and plasma membrane integrity to prevent apoptosis.

#### 7.2.8. Histone Deacetylases Inhibitors

Histone deacetylases inhibitors (HDACi) mediate the catalytic activity of histone deacetylases (HDACs) by targeting the lysine residues on histone and non-histone proteins. These inhibitors alter the acetylation status of proteins involved in the regulation of oxidative stress, cell proliferation and apoptosis [165,166].

In FRDA, the presence of HDACs and absence of histone acetylation are responsible for FXN gene silencing and frataxin deficiency [167]. HDACi has been revealed to reverse heterochromatin-mediated FXN silencing by increasing mRNA expression and protein level of frataxin and promoting histone hyperacetylation in lymphocytes derived from patients with FRDA [168,169,170].

HDACi contains a benzamide functional group targeting HDAC1/2 and HDAC3, in which HDAC1/2 form the core catalytic components of co-repressor complexes that modulate gene expression, whereas HDAC3 contributes to the regulation of gene expression, chromatin structure and genomic stability [170]. Codazzi et al. [128] investigated the protective effects of benzamide HDACi-109 (N-(6-(2-aminophenylamino)-6-oxohexyl)-4-methylbenzamide pimelic diphenylamide) on iPSCs derived from patients with FRDA. Administration of 5 to 10 µM HDACi-109 attenuated mitochondrial SOD2, implicating a compensatory response to chronic elevation in mitochondrial ROS. Further investigation showed that the ability of HDACi in counteracting oxidative stress was associated with elevated protein level of frataxin, scaffold proteins (ISCUs), FeS apoprotein (aconitase 2 and NDUFS3), and lipoic acid-containing proteins, the OGDH and PDH, indicating the restoration of FeS bioavailability in iPSCs.

#### 7.2.9. Kinetin

Kinetin (N6-furfuryladenine) is a small-molecule adenosine and an affordable substitute for natural cytokinin in plant tissue culture. Kinetin has been found to successfully ameliorate neurodegeneration in Parkinson’s and Huntington’s diseases [171,172].

Maková et al. [129] reported the protective effects of synthetic kinetin bioisosteres against BSO-induced oxidative stress in fibroblasts derived from patients with FRDA. Synthesis of the novel compounds was achieved by replacing the purine ring with other bicyclic heterocycles. Administration of 200 μM 1N-(furan-2-ylmethyl)-1H-imidazo [4,5-*c*]pyridin-4-amine (compound **5**) slightly activated FeS clusters biogenesis, whereas 6-chloro-N-(furan-2-ylmethyl)-1H-imidazo [4,5-*c*]pyridin-4-amine (compound **6**) slightly decreased FeS clusters biogenesis, indicating the role of these compounds in the protection against secondary effects of frataxin deficiency rather than substitution of frataxin in a reconstituted FeS machinery lacking frataxin.

#### 7.2.10. Liver Growth Factor

The liver growth factor (LGF) is a hepatic mitogen and albumin–bilirubin complex that exhibits remarkable protective effects in preclinical models of hepatic and extrahepatic diseases [173,174], and neurodegenerative diseases [175,176,177].

Intraperitoneal injection of 1.7 μg LGF for three weeks improved motor coordination assessed by rotarod test, demonstrated neuroprotection in lumbar region and reversed cardiac hypertrophy in a YG8R mouse model mediated by increased protein level of frataxin [130]. LGF inhibited neuronal apoptosis in the lumbar region by increasing NeuN expression and restored complex IV and cytochrome *c* levels in the spinal cord, and heart and CNS tissues, respectively. The ratios of phospho-Akt/Akt and Bcl2/Bax were increased in the spinal cord and in the brainstem and cerebellum, respectively, which further confirmed the role of pro- and anti-apoptotic proteins in mediating neuronal survival. In addition, LGF attenuated oxidative stress in the skeletal muscle by decreasing GSSG levels and increasing GSH levels, indicating restoration of redox homeostasis.

#### 7.2.11. Methylene Blue and Methylene Violet

Methylthioninium chloride, also known as methylene blue (MB), is a cationic colorant belonging to the family of phenothiazines. At present, MB is the first line of treatment in methemoglobinemia and ifosfamide-induced encephalopathy through its inhibitory activity against monoamine oxidase [178]. Preclinical studies have revealed the therapeutic potential of MB in Alzheimer’s disease [179,180], stroke, global cerebral ischemia, Parkinson’s disease and traumatic brain injury [180].

Administration of 2.5 µM MB analogs have been shown to prevent BSO- and rotenone-induced oxidative stress in fibroblasts and lymphocytes derived from patients with FRDA, respectively [131]. The analogs attenuated accumulation of ROS, increased the protein level of frataxin in the regulation of FeS clusters and mitochondrial biogenesis, enhanced aconitase activity, restored the mitochondrial function by promoting ATP production and activity of complex I, and inhibited MMP dissipation. MB functions as an alternative electron carrier by shuttling electrons between NADH and cytochrome *c* and therefore creating a mechanism of bypassing the complexes I and III. In this manner, MB is insensitive to rotenone, a specific inhibitor of complex I, and can thus reduce electron leakage from ETC and regulate continuous ATP production.

Subsequently, Khdour et al. [132] demonstrated the hydrolysis of MB in the formation of hydrophobic methylene violet (MV) under highly basic conditions. In its naturally occurring oxidized (quinone) form, MB can be reduced at the mitochondrial redox centers, generating the phenolic (quinol) form of antioxidant, analogous to CoQ_10_. MV analogs possessed stronger protective effects against diethyl maleate-induced oxidative stress compared to the MB analogs in lymphoblasts derived from patients with FRDA. Khdour et al. [132] observed that 0.1 μM to 2.5 μM MB and MV analogs increased ATP production and prevented MMP dissipation in lymphoblasts derived from patients with FRDA. Additionally, the analogs increased the protein level of frataxin and aconitase activity, as well as promoted mitochondrial biogenesis, regulated by succinate dehydrogenase (SDH-A), a subunit of complex II and cytochrome *c* oxidase subunit 1 (COX1), a subunit of complex IV.

In a study by Roy Chowdhury et al. [133], five lipophilic MV analogs were investigated in fibroblasts and lymphocytes derived from patients with FRDA. Administration of 250 to 2500 nM MV analogs protected fibroblasts and lymphocytes against BSO- and rotenone-induced oxidative stress, respectively, by attenuating the accumulation of ROS. In addition, MV analogs improved mitochondrial function through enhancement of intracellular ATP production and activity of NADH-ubiquinone oxidoreductase (complex I), and restoration of MMP dissipation. Intriguingly, two of the analogs with longer alkyl side chains showed greater potency in attenuating intracellular ROS and apoptosis compared to their counterparts [133].

Liu et al. [134] further confirmed the protective effects of MV and MB analogs ranging from 100 nM to 2.5 μM against erastin-induced cell death in primary fibroblasts and RAS-selective lethal 3 (RSL-3)-induced lipid peroxidation in lymphocytes derived from FRDA patients. MV analogs designated as 1b–5b have been shown to exhibit stronger protective effects against erastin- and RSL-3-induced oxidative stress compared to the parent molecule of phenothiazine by inhibiting ferroptosis and promoting mitochondrial biogenesis. The anti-ferroptotic activity of the analogs was regulated by AMP-activated protein kinase (AMPK), a sensor of cellular energy status, represented by an increase in p-AMPK/AMPK ratio. Moreover, these analogs were more potent than the inhibitors of ferroptosis, namely ferrostatin-1 (Fer-1), liproxstatin-1 (Lip-1) and α-tocopherol (α-TOH).

#### 7.2.12. N-Acetylcysteine

N-acetylcysteine (NAC) is an aminothiol and synthetic precursor of intracellular cysteine and GSH. The therapeutic role of NAC as a potent antioxidant is directly linked to its ability to regulate the intracellular level of cysteine with a subsequent increase in GSH. Oral administration of NAC, which replenishes the cysteine required for GSH synthesis, has been tested in a large number of randomized placebo-controlled trials involving oxidative-stress diseases related to GSH deficiency [181].

Petrillo et al. [117] revealed the therapeutic effects of NAC in the modulation of the NRF2 signaling pathway in fibroblasts derived from patients with FRDA. Administration of 100 µM NAC promoted mRNA expression and protein levels of NRF2 and its downstream targets, namely HO-1, NQO1 and GCL, leading to increased expression of FXN. Furthermore, NAC upregulated the protein level of DJ-1, a stabilizer of NRF2, enabling translocation of NRF2 from cytoplasm to the nucleus and upregulating transcription of antioxidant and oxidative stress response genes by preventing its binding to KEAP1.

#### 7.2.13. Oleic Acid

Oleic acid (OA) is a monounsaturated omega-9 fatty acid that is found abundantly in olive oil, accounting for 70–80% of its composition. It is also a nonessential fatty acid responsible for the regulation of immune function and its substitution to dietary saturated fat has been revealed to reduce the risks of cardiovascular diseases [182].

Cotticelli et al. [135] demonstrated the therapeutic effects of 20 μM OA in the prevention of erastin-induced ferroptosis in murine fibroblasts harboring frataxin-associated I154F point mutation. Erastin was first discovered as an inducer of iron-dependent cell death accompanied by antioxidant depletion caused by cystine glutamate antiporter inhibition. Additionally, a series of fatty acids and fatty-acid analogs at 40 μM, namely methyl ester, ethyl ester, amide and hydroxamic acid, have been found to protect against ferric ammonium citrate (FAC)- and L-buthionine (S,R)-sulfoximine (BSO)-induced cytotoxicity in the murine fibroblasts. FAC and BSO inhibit the rate-limiting step in GSH biosynthesis. Trifluoromethyl ketones analogs (derivatives of oleic acid), namely *(R)*-24 and *(S)*-24, ranging from 1 to 40 μM, rescued TERT-immortalized fibroblasts from FAC- and BSO-induced cytotoxicity, whereas 5 μM *(R)*-24 protected the fibroblasts cell lines from erastin-induced ferroptosis. Further, *(R)*-24 and *(S)*-24 also inhibited lipid peroxidation in cultured myoblasts with FXN knock-down (siFXN-1) in the presence of RSL-3, a ferroptosis inducer.

#### 7.2.14. Omaveloxolone

Omaveloxolone (Omav), a synthetic oleanane triterpenoid compound, has been shown to alleviate oxidative stress in a broad range of diseases by restoring depleted endogenous antioxidants [102,183,184,185]. The protective effects of Omav were attributed to the activation of NRF2 through the silencing of KEAP-1 expression [102].

Abeti et al. [136] examined the therapeutic effects of Omav in fibroblasts derived from patients with FRDA, in granule neurons of cerebellums (CGNs) derived from FXN KIKO mice, and in GAA-expanded transgenic Y8GR mice. Administration of 50 nM Omav prevented the inhibition of complex I by increasing the NADH pool and decreasing the NADH redox state in FRDA fibroblasts exposed to H_2_O_2_ and in CGNs. Subsequent analysis showed that Omav restored GSH levels and attenuated the increased susceptibility to lipid peroxidation and mitochondrial ROS through NRF2 and KEAP1. Furthermore, Omav conferred protection against apoptosis-associated mitochondrial dysfunction and restored MMP dissipation.

Petrillo et al. [117] also investigated the role of Omav in mediating the NRF2 signaling pathway in fibroblasts derived from patients with FRDA. Administration of 100 nM Omav increased mRNA expression and protein levels of NRF2 and its downstream genes, namely NQO1, HO-1 and GCL. Furthermore, Omav also increased the level of GSH, suggesting its ability to exert antioxidant activities by regulating NRF2 and KEAP1-DJ-1-p62 signaling pathways.

#### 7.2.15. Peroxisome Proliferator-Activated Receptor Gamma Agonist

The peroxisome proliferator-activated receptor-γ (PPARγ) coactivator 1 alpha (PGC-1α), is an integrative transcriptional regulator of oxidative metabolism, including fatty acid oxidation and mitochondrial biogenesis [186,187]. Frataxin deficiency has been found to cause dysregulation of the PPARγ/PGC-1α-signaling pathway, evidenced by downregulation in the transcriptional activity of PGC-1α in FXN KIKO and KIKI mice models and fibroblasts derived from patients with FRDA. Leriglitazone, a PPARγ agonist, has been found to increase the protein level of frataxin and PGC-1α and its downstream target, glucose-regulated protein 75 (GRP75), in fibroblasts derived from patients with FRDA, contributing to restoration of mitochondrial function and biogenesis [137]. GRP75 is a mitochondrial molecular chaperone responsible for the regulation of FeS cluster biogenesis and mitochondrial homeostasis.

Similarly, Dong et al. [138] postulated that interaction between GRP75 and mitochondrial processing peptidase (MPP) in cortical homogenates, cultured primary cortical neurons and human embryonic kidney 293 (HEK293) cells co-transfected with frataxin and hemagglutinin-tagged ubiquitin (HA-Ub) enhanced the accessibility of frataxin to MPP. An interaction between GRP75 and frataxin forms a complex with mitochondrial ISCU2, the core of FeS clusters. Moreover, GRP75 has been observed to regulate frataxin and therefore an overexpression of GRP75 restores frataxin deficiency and ATP level in fibroblasts with siRNA knockdown of frataxin and in HEK 293 cells. The ISCU2 has been suggested to compensate for the mitochondrial defects, including abnormal network.

Additionally, Rodríguez-Pascau et al. [137] revealed the protective effects of leriglitazone in rat DRG sensory neurons transduced with lentiviral vector expressing short hairpin RNA (shRNA) for silencing of FXN1 and in YG8sR mice. Frataxin deficiency induces neuronal swelling and formation of neurofilament aggregates, leading to neuronal degeneration. Administration of 500 nM leriglitazone has been found to attenuate the formation of neurofilament aggregates and improve mitochondrial function and calcium homeostasis. Restoration of Ca^2+^ signaling and NCLX, a mitochondrial Na^+^/Ca2^+^ exchanger, increased protein level of frataxin and reduced calpain, and caspase 3 mediated-cleavage of α-fodrin promoted the survival of sensory neurons, therefore strengthening the hypothesis of a central role for calcium homeostasis in frataxin-deficient DRG. Furthermore, oral administration of 50 mg/kg has been revealed to improve motor performance in YG8sR mice, assessed by rotarod, pole and balance beam tests.

#### 7.2.16. Sulforaphane

Sulforaphane (SFN) belongs to the isothiocyanate group of organosulfur compounds. Recent evidence has suggested the involvement of epigenetic mechanisms of SFN in the regulation of NRF2-mediated gene expression through the inhibition of HDACs [117,188,189,190,191].

In a study by Petrillo et al. [139], SFN was investigated for its antioxidant activities in cultured FXN-silenced NSC34 motor neurons and fibroblasts derived from patients with FRDA. Administration of 5 µM SFN showed upregulation of mRNA expression and protein levels of NRF2 and its downstream targets of Phase II antioxidant enzymes, namely heme oxygenase-1 (HO-1), NQO1, copper, zinc superoxide dismutase (Cu/Zn SOD), SOD1, SOD2, GCLC and GCLM, initiating reorganization of network formation and stimulation of neurite outgrowth. Further, SFN has been found to decrease the level of GSSG and increase the level of reduced GSH, indicating restoration of redox homeostasis in FXN-silenced motor neurons, as well as consistently enhance the level of frataxin in shFXN motor neurons. Additionally, Petrillo et al. [117] have shown that regulation of KEAP1-DJ-1-p62 activates the NRF2-mediated antioxidant pathway in fibroblasts derived from patients with FRDA, leading to increased GSH content, mRNA expression and protein level of NQO1, HO-1 and GCL. The observation was associated with a robust increase in the expression of FXN mRNA [117,139].

On the other hand, La Rosa et al. [119] investigated the effects of SFN on pre-symptomatic NRF2 impairment in an in vitro model of FRDA, using NSCs derived from FXN KIKO mice. Administration of 5 µM SFN increased mRNA expression and protein level of NRF2 and its downstream targets (NQO1 and HO-1), leading to restoration of antioxidant defense systems and reduced ROS accumulation in NSCs. Additionally, NRF2 activation contributed to the recovery of neuronal morphology and differentiation as well as prevented the phenotypic defects of NSCs.

Ferroptosis is an iron-dependent cell death caused by iron-mediated lipid peroxidation with accumulation of lipid peroxidation products, elevation of ROS level, depletion of GSH and an increase in iron bioavailability [192]. Ferroptosis inhibitors targeting pathways involved in ferroptosis execution have been proposed as a therapeutic application for the management of FRDA [193]. A follow-up study by La Rosa et al. [118] reported the ability of SFN to prevent ferroptosis in whole blood and fibroblasts derived from patients with FRDA, and in FXN KIKO mice. Administration of 10 µM SFN decreased lipid peroxidation and rescued the morphological defects of the mitochondria, suggesting restoration of redox homeostasis modulated by ferroptosis-related genes, namely the FXN, NRF2, SOD2, GPX4 and GCL.

**Figure 8:**
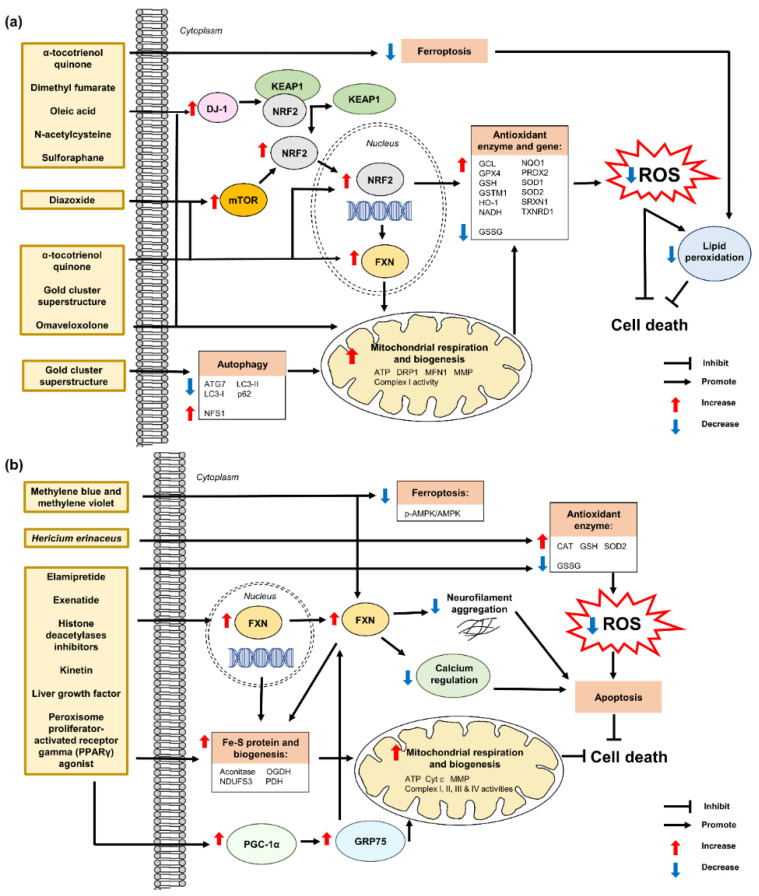
Antioxidant therapies against FRDA. (**a**) Regulation of KEAP1–NRF2 complex, mTOR expression, lipid peroxidation, mitochondrial impairment and autophagy by antioxidant therapies results in the dissociation of KEAP1–NRF2 complex, restoration of mitochondrial respiration and biogenesis, and attenuation of lipid peroxidation. The dissociation of the KEAP1–NRF2 complex leads to the upregulation of downstream targets responsible for increased levels of antioxidant enzyme and gene, and attenuation of ROS production and pro-apoptotic activities. (**b**) Regulation of FXN expression and antioxidant enzymes by antioxidant therapies results in the promotion of FeS clusters biogenesis and restoration of mitochondrial function. Increased protein level of frataxin restores calcium regulation and attenuates neurofilament aggregate formation. The events ultimately lead to the attenuation of ROS production and pro-apoptotic activities. AMPK, AMP-activated protein kinase; ATG7, autophagy related 7; ATP, adenosine triphosphate; CAT, catalase; Cyt *c*, cytochrome *c*; DJ-1, protein deglycase; DRP1, dynamin-related protein 1; FXN, frataxin; GCL, γ-glutamyl cysteine ligase; GPX4, glutathione peroxidase 4; GRP75, glucose-regulated protein 75; GSH, glutathione; GSSG, oxidized glutathione; GSTM1, glutathione S-transferase mu 1; HO-1, heme oxygenase-1; KEAP1, Kelch-like ECH-associated protein 1; LC3, microtubule-associated protein 1A/1B-light chain 3; MFN1, mitofusin 1; MMP mitochondrial membrane potential; mTOR, mammalian target of rapamycin; NDUFS3, NADH: ubiquinone oxidoreductase core subunit s3; NFS1, cysteine desulfurase; NOQ1, NAD(P)H quinone oxidoreductase 1; NRF2, nuclear factor erythroid 2–related factor 2; NSF, N-ethylmaleimide-sensitive fusion protein; OGDH, 8-oxoglutarate dehydrogenase E1 component; PDH, pyruvate dehydrogenase; PGC-1α, peroxisome proliferator-activated receptor-γ (PPARγ) coactivator 1 alpha; PRDX2, peroxiredoxin 2; ROS, reactive oxygen species; SOD, superoxide dismutase; SRXN1, sulfiredoxin; TXNRD1, thioredoxin reductase 1.

Figure 8 shows a schematic of all the modes of action of current antioxidant therapies for the management of FRDA.

## 8. Limitations and Perspectives for Future Research

To date, no effective therapies have been proposed for ARCAs, even if some evidence suggests that powerful antioxidant agents can be considered as a therapeutic tool. Considering that mitochondrial dysfunction often correlates to excessive ROS production, many therapeutic approaches envisage the exploitation of antioxidants. Nevertheless, the discovery of effective therapy to address the multisystemic issues of ARCAs has been challenging because it requires well-established clinical trials with a statistically sufficient number of patients and preclinical experiments with minimal confounding factors, accurate biomarkers, and appropriate cellular or animal models. Given that ARCAs cause systemic and progressive symptoms, a multidisciplinary approach is often engaged to relieve these symptoms.

Although many potential new drugs or therapeutic strategies have been investigated for the management or to cure ARCAs, from gene-based therapies to small molecules and peptides, from novel monoclonal antibodies to engineered cell-based therapies, the majority of them have only been tested in a small sample size of cohort studies of Phase I and/or Phase II clinical trials or in preclinical models that do not fully recapitulate the multisystemic nature of the disease. For instance, Omav and DMF demonstrated promising results in cellular models of FRDA by increasing the mRNA expression of frataxin and the levels of antioxidant defense markers [117,122]. However, Lynch et al. [194] revealed that Omav can cause upper respiratory tract infections and nasopharyngitis observed in a Phase II clinical trial. To overcome these challenges, Galeano et al. [195] proposed a machine-learning approach for predicting the frequencies of adverse effects of drugs, indicating the model is highly reliable in providing valuable information about the biological events underlying their activities at the anatomical and molecular levels.

Complementary and alternative medicines have been used for decades to treat various diseases. Synergistic therapeutic effects have been mainly demonstrated in herbal formulations and TCM [11,196]. The safety and effectiveness of some of these formulations have been questioned because of the proprietary formulation used and there have been no well-designed pharmacovigilance studies of these formulations. Due to the high abundance of non-desirable components, bioassay-guided isolation of target compounds in the formulations must be performed with subsequent identification [197]. Metal impurities, fluorescence-interfering, and the presence of non-polar or polar compounds in the formulations may provide inaccurate results for some bioassays and therefore must first be eliminated to avoid interference [198]. Nevertheless, emerging trends in nanotechnology are revolutionizing the development of natural antioxidants. Nanotechnology can be used to facilitate the delivery of natural antioxidant compounds by delaying the development of drug resistance, with improved responses comparable to modern medicine approaches. In this respect, nanotherapeutics could be developed to facilitate the delivery of these compounds for ARCAs by enhancing bioactivity, improving bioavailability at the target sites and allowing sustained drug release with prolonged action [199]. For instance, nanosensors have been proposed as an emerging tool in the study of the modulation of oxidative stress and individual responses to drugs in FRDA [200].

The extent of involvement of oxidative stress in the pathogenesis of ARCAs has been controversial. Uceda et al. [201] highlighted the complex molecular mechanism caused by frataxin deficiency in FRDA. Indeed, oxidative stress causes extensive alterations in the structures of DNA, including the base and sugar lesions, DNA–protein cross-links, strand breaks and base-free sites. Impaired mitochondrial function, DNA repair efficiency, synaptic transmission, chaperone activity and metabolic functioning are the common pathological mechanisms across ARCAs. Therefore, it is important to understand the mapping of the crosstalk between these major pathways in mediating the progression of ARCAs. The distinctive features of ARCAs models, vulnerability of different cellular models and animal tissues against various inducers of oxidative stress and time points of analysis could partially explain some of the apparently contradictory results [117,118,119,139,202].

Besides, another factor that hinders the development of therapeutics in ARCAs is the conflicting research findings. In FRDA, idebenone may be used for regressing left ventricular hypertrophy to a certain extent; however, the ejection fraction failed to stipulate any improvement in cardiac function [203]. Other than that, Singh et al. [204], Lee et al. [205] and Chiang et al. [202] revealed that SFN depleted the level of GSH, promoted excessive production of ROS and mitochondrial impairment, and induced cell death in FRDA models. This is in contrast to the findings of recent studies by Petrillo et al. [117,139] and La Rosa et al. [118,119]. SFN has been shown to increase the level of GSH and attenuate lipid peroxidation and mitochondrial ROS production in preclinical models of FRDA, suggesting there is insufficient evidence to support or refute a benefit of SFN for treatment of ARCAs. A combination of various determining and confounding factors such as regression to the mean, environmental differences between laboratories and methodological choices can contribute to the generation of discrepant analyses and results.

While the prevalence of hereditary ataxia is well established in many Western countries, data on ARCAs in southeast Asia is lacking. At present, there is no further data on ARCAs within the region apart from two published studies that identified the consequences of a deletion of novel dinucleotide in the first exon of FXN gene and deficiency of complex I, causing the pathogenesis of FRDA [206,207]. Nevertheless, these studies can be applied to large-scale epidemiological study on ARCAs in this region. There is a need for collaborative efforts among experts to investigate the challenges and priorities in the detection and management of ARCAs in this region. A successful planning and execution of a research consortium involving the Movement Disorder Society (MDS), National Organization for Rare Disorders (NORD), Friedreich’s Ataxia Research Alliance (FARA) and National Ataxia Foundation (NAF) raises the exciting possibility for ARCAs research. Discovery of novel neuroimaging biomarkers that could facilitate future diagnosis and appropriate patient registry are key areas in enhancing comprehensive management of patients with ARCAs.

The development of appropriate animal models for ARCAs research will be instrumental in facilitating our understanding of these diseases. The translation of promising preclinical findings to positive clinical outcomes often disappoints because of discrepancies in the complexity and differences between model species as well as the heterogeneous response to the same treatment in different patients. Genetically engineered animals and animals with spontaneous mutations have been utilized in the development of innovative treatments for many rare and incurable diseases [208,209]. However, animal models can be limited in revealing fundamental aspects of human disease such as pathogenesis, genetics, and mechanisms. The value of using animals to predict the effectiveness of treatment strategies in clinical trials has remained controversial. The failure of many clinical trials based on animal studies, particularly rodents, has further highlighted the limitations of animal-based research [210]. Collectively, this could lead to failures in targeted therapeutic interventions in affected individuals. To determine the full therapeutic potential of a candidate antioxidant compound, preclinical studies must examine their effects in terms of the molecular profile of ARCAs, as well as mobility and neurological testing using appropriate in vivo animal models that exhibit the various age-dependent symptoms experienced by patients with ARCAs before progressing to clinical trials.

## 9. Conclusions

Taken together, this systematic review provides a promising look at the current research on antioxidant therapeutics for ARCAs. A better understanding of clinical and neuropathological features and the genes associated with the complex phenotypes may lead to advances in therapeutic strategies, including novel cell therapy, mitochondria-targeted antioxidants and cell-permeable peptide antioxidants. Nevertheless, the safety and efficacy of formulations continues to be a major issue due to the lack of proper pharmacovigilance studies, despite their expected lower risk compared with synthetic drugs. Issues of conflicting findings between studies may deter the future progress of these novel therapeutics. There are significant gaps between the resources needed for research and those that are currently available, and a pressing need to strengthen institutional capacity and research infrastructure while promoting both local and regional research collaborations. Therefore, it is crucial that future studies implement suitable tools and models of ARCAs to accelerate the translation to clinical practice.

## Figures and Tables

**Table 1:** Antioxidant therapies in the management of ataxia-telangiectasia.

Therapy	Model	Finding	Mode of Action	Reference
α-lipoic acid 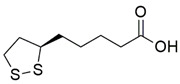	IL-1β-induced oxidative stress in human fibroblasts	Attenuation of ROS production Restoration of mitochondrial function	↓ mRNA expression and protein level of IL-8 ↓ NF-kB activation	[26]
CRISPR/Cas9	H_2_O_2_-induced oxidative stress and 2DG-induced metabolic stress in human iPSCs	Restoration of DNA damage response, cell cycle control and ATM kinase	↑ KAP1 ↓ γ-H2A.X and cleaved caspase 3	[103]
Dexamethasone 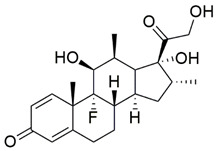	Human lymphoblasts	Attenuation of ROS production ↑ reduced GSH and total GSH	↑ mRNA expression of GCLC, GCLM, GSS and GSR ↑ mRNA expression and protein level of NRF2	[104]
	Human lymphoblasts	Activation of NRF2	↓ mRNA expression and protein level of KEAP1	[22]
Genetic suppressor element 4	Human fibroblasts and lymphoblasts	Protection against apoptosis Attenuation of oxidative stress-induced DNA damage Reconstitution of telomerase activity and elongation	↑ OGG1 ↓ mRNA expression of IL-6, SOD1 and TERT ↓ 8-oxoG and p38 phosphorylation	[105]

**Table 2:** Antioxidant therapies in the management of Friedreich’s ataxia.

Therapy	Model	Finding	Mode of Action	Reference
α-tocotrienol quinone 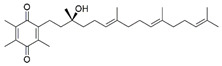	Human fibroblasts	Modulation of NRF2	↑ mRNA expression and protein levels of frataxin, NRF2, NQO1, HO-1 and GCL Regulation of KEAP1-DJ-1-p62	[117]
	Human fibroblasts	Protection against ferroptosis Attenuation of lipid peroxidation Restoration of mitochondrial function	↑ mRNA expression of FXN, SOD2 and GPX4, GCL ↑ mRNA expression and protein level of NRF2	[118]
	NSCs derived from FXN KIKO mice	Attenuation of ROS production Restoration of morphology, differentiation and phenotypic defects	↑ mRNA expression and protein levels of NRF2, NQO1 and HO-1	[119]
Diazoxide 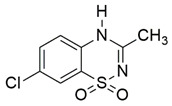	Human lymphoblasts	Protection against oxidative stress	↑ mRNA expression and protein level of frataxin ↑ mTOR-S6K and nuclear translocation of NRF2	[120]
	YG8sR mice	↑ fine motor coordination and balance, and stride length ↑ aconitase ↓protein oxidation in brain, liver and pancreas	↑ mRNA expression and protein level of frataxin in cerebellum and heart ↑ mRNA expression of NRF2 in cerebellum and heart	
Dimethyl fumarate 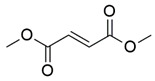	Human fibroblasts	Modulation of NRF2 ↑ GSH	↑ mRNA expression and protein levels of frataxin, NRF2, NQO1, HO-1 and GCL Regulation of KEAP1-DJ-1-p62	[117]
	Human fibroblasts and blood FXN KIKO mice	↑ mitochondrial biogenesis	↑ mRNA expression and protein of frataxin ↑ mRNA expression of NRF1 and mTFA	[121]
	Human fibroblasts	↑ mitochondrial biogenesis	↑ mRNA expression and protein level of frataxin	[122]
	Human lymphoblasts YG8 and FXN KIKO mice	↑ protein level of frataxin	↑ mRNA expression of frataxin ↓ R-loop formation and transcriptional silencing restricted FXN locus	
Elamipretide 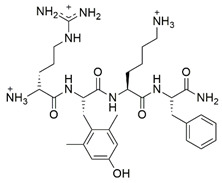	Human fibroblasts and lymphoblasts	Attenuation of ROS production Restoration of MMP and mitochondrial morphology ↑ aconitase, complex II and III, SOD and CAT	↑ frataxin, ATP and NAD+/NADH	[123]
	Y47 and YG8R mice	↑ motor function ↓ cytoplasmic vacuolization in DRG and lesions in the dentate nuclei Restoration of damaged myelin in the spinal cord	↑ mRNA expression of frataxin	[124]
Exenatide 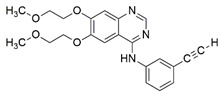	Human iPSC-derived β cells and sensory neurons	Restoration of mitochondrial function	↑ frataxin, aconitase, NDUFS3, OGDH and PDH	[125]
	FXN KIKO mice	↑ glucose tolerance, β cell function and insulin secretion Neuroprotection against DRG	↑ protein level of frataxin and ISC-containing protein ferrochelatase in cerebellum and cerebrum	
Gold cluster superstructure	Human MSCs	Attenuation of ROS production Restoration of mitochondrial function and bioenergetic capacity, ATP, ETC function and MMP dissipation	↑ frataxin Modulation of autophagic flux, frataxin-related proteins and dynamin-related proteins	[126]
	YG8sR mice	Restoration of motor deficits, neuromuscular function, cardiac contractility, mitochondrial and ETC function Attenuation of ROS production ↑ ATP ↓ collagen deposition in the skeletal muscle and cardiac fibrosis	↑ NSF and PGC-1α ↓ 4-HNE, 8-oxodG, LC3-II/LC3-1 and PPARγ Activation of NRF2-ARE	
*Hericium erinaceus*	BSO-induced oxidative stress in human fibroblasts	Restoration of GSH/GSSG and plasma membrane integrity Prevention of apoptosis	NE	[127]
Histone deacetylases inhibitors 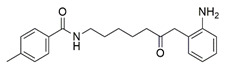	Human iPSCs	Protection against oxidative stress	↑ frataxin, ISCUs, aconitase 2, NDUFS3, OGDH and PDH ↓ ROS and SOD2	[128]
Kinetin 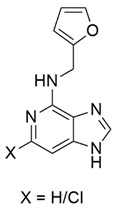	BSO-induced oxidative stress in human fibroblasts	Protection against secondary effects of frataxin deficiency	NE	[129]
Liver growth factor	YG8R mice	Restoration of motor coordination Attenuation of neuronal apoptosis Reversal of cardiac hypertrophy ↑ GSH ↓ GSSG	↑ frataxin, complex IV and cytochrome *c* ↑ phospho-Akt/Akt and Bcl2/Bax	[130]
Methylene blue analogs 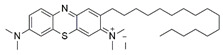	BSO-induced oxidative stress in human fibroblasts Rotenone-induced oxidative stress in human lymphocytes	Attenuation of ROS production Restoration of mitochondrial function and biogenesis ↑ aconitase, ATP and MMP	↑ frataxin and complex I	[131]
Methylene violet analogs Compound **1** 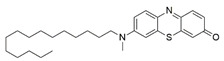 Compound **2** 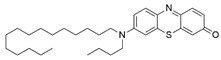 Compound **4b** 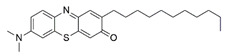 Compound **6b** 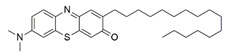	Diethyl maleate-induced oxidative stress in human lymphoblasts	Restoration of mitochondrial biogenesis ↑ aconitase, ATP and MMP	↑ frataxin Regulation of SDH-A and COX-1	[132]
Methylene violet analogs Compound **4** 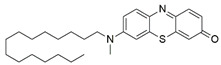 Compound **5** 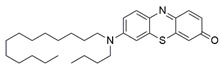	BSO-induced oxidative stress in human fibroblasts Rotenone-induced oxidative stress in human lymphocytes	Attenuation of ROS production Restoration of mitochondrial function ↑ ATP and MMP	↑ NADH:ubiquinone oxidoreductase (complex I)	[133]
Methylene violet analogs Compound **1b** 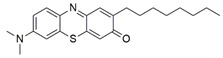 Compound **2b** 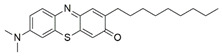 Compound **3b** 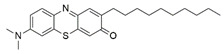 Compound **4b** 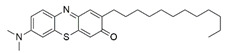 Compound **5b** 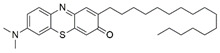	Erastin-induced oxidative stress in human fibroblasts RSL3-induced oxidative stress in human lymphocytes	Protection against ferroptosis Restoration of mitochondrial biogenesis	Regulation of AMPK ↑ pAMPK/AMPK	[134]
N-acetylcysteine 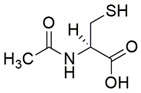	Human fibroblasts	Modulation of NRF2	↑ mRNA expression and protein levels of frataxin, NRF2, NQO1, HO-1 and GCL Regulation of KEAP1-DJ-1-p62	[117]
Oleic acid 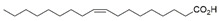	Erastin-induced ferroptosis in murine fibroblasts	Protection against ferroptosis	NE	[135]
Fatty acids and fatty-acid analogs Oleic acid derivatives 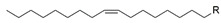 Cis-Vaccenic acid (7) 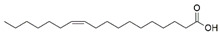 Petroselinic acid (8) 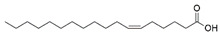 Gadoleic acid (10) 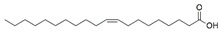 Erucic acid (11) 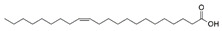 Heptadecenoic acid (12) 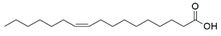 Palmitoleic acid (13) 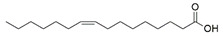	FAC- and BSO-induced oxidative stress in murine fibroblasts	Protection against cytotoxicity	NE	
*(R)*-24 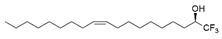 *(S)*-24 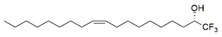	FAC- and BSO-induced oxidative stress in human TERT-immortalized fibroblasts RSL-3-induced oxidative stress in siFXN-1 myoblast	Protection against cytotoxicity Protection against ferroptosis	NE	
*(R)*-24 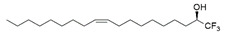	Erastin-induced ferroptosis in human fibroblasts	Protection against ferroptosis	NE	
Omaveloxolone 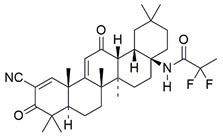	H_2_O_2_-induced oxidative stress in human fibroblasts FXN KIKO and Y8GR mice	Prevention of complex I inhibition Attenuation of ROS production and lipid peroxidation Restoration of GSH, mitochondrial function and MMP dissipation	↑ mRNA expression and protein level of NRF2 ↑ NADH pool ↓ NADH redox state Regulation of KEAP1	[136]
	Human fibroblasts	Modulation of NRF2 ↑ GSH	↑ mRNA expression and protein level of NRF2, NQO1, HO-1 and GCL Regulation of KEAP1-DJ-1-p62	[117]
Peroxisome proliferator-activated receptor gamma agonist Leriglitazone 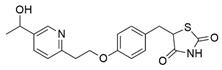	Human fibroblasts	Restoration of mitochondrial function and biogenesis	↑ frataxin, PGC-1α and GRP75	[137]
	DRG sensory neurons	Attenuation of formation of neurofilament aggregates Restoration of mitochondrial function and calcium homeostasis	↑ frataxin and NCLX ↓ cleavage of α-fodrin	
	YG8sR mice	Restoration of motor function	NE	
Peroxisome proliferator-activated receptor gamma agonist GRP75	Cortical homogenates, primary cortical neurons and HEK293 cells	↑ accessibility of frataxin to MPP	↑ ISCU2	[138]
	Human fibroblasts and HEK293	Restoration of frataxin, mitochondrial network and ATP	↑ frataxin and ISCU2	
Sulforaphane 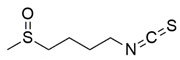	FXN-silenced NSC34 motor neurons	↑ reduced GSH ↓ GSSG Reorganization of network formation and stimulation of neurite outgrowth	↑ mRNA expression and protein levels of frataxin, NRF2, NQO1, NQO1, Cu/Zn SOD, SOD1, SOD2, GCL-C and GCL-M	[139]
	Human fibroblasts	Modulation of NRF2 ↑ GSH	↑ mRNA expression and protein levels of frataxin, NRF2, NQO1, HO-1 and GCL Regulation of KEAP1-DJ-1-p62	[117]
	NSCs derived from FXN KIKO mice	Attenuation of ROS production Restoration of morphology, differentiation and phenotypic defects	↑ mRNA expression and protein levels of NRF2, NQO1 and HO-1	[119]
	Human fibroblasts and blood FXN KIKO mice	Protection against ferroptosis Attenuation of lipid peroxidation Regulation of mitochondrial morphology	↑ mRNA expression of FXN, SOD2, GPX4 and GCL ↑ mRNA expression and protein level of NRF2	[118]

## Data Availability

Data is contained within the article.

## Abbreviation

ATM	Ataxia-telangiectasia mutated;
DNA	deoxyribonucleic acid;
GCLC	glutamyl-cysteine ligase catalytic subunit;
GCLM	glutamyl-cysteine ligase modifier subunit;
GRE	genetic suppressor element;
GSH	glutathione;
GSR	glutathione reductase;
GSS	glutathione synthetase;
IL	interleukin;
iPSCs	induced pluripotent stem cells;
KAP1	KRAB-associated protein 1;
KEAP1	Kelch-like ECH-associated protein 1;
mRNA	messenger ribonucleic acid;
NF-kB	nuclear factor kappa-light-chain-enhancer of activated B cells;
NRF2	nuclear factor erythroid 2–related factor 2;
OGG1	8-oxoguanine DNA glycosylase-1;
ROS	reactive oxygen species;
SOD	superoxide dismutase;
TERT	telomerase reverse transcriptase;
γ-H2A.X	gamma-H2A histone family member X;
2DG	deoxy-D-glucose;
8-oxoG	8-oxoguanineAkt, protein kinase B;
AMPK	AMP-activated protein kinase;
ARE	antioxidant response element;
ATP	adenosine triphosphate;
Bax	Bcl2 associated X;
Bcl-2	B-cell lymphoma 2;
BSO	L-buthionine (S,R)-sulfoximine;
CAT	catalase;
COX1	cytochrome *c* oxidase I;
DRG	dorsal root ganglion;
FAC	ferric ammonium citrate;
FXN	frataxin;
GCL	γ-glutamyl cysteine ligase;
GCLC	glutamyl-cysteine ligase catalytic subunit;
GCLM	glutamyl-cysteine ligase modifier subunit;
GPX4	glutathione peroxidase 4;
GRP75	glucose-regulated protein 75;
GSH	glutathione;
GSSG	oxidised glutathione;
HO-1	heme oxygenase 1;
iPSCs	induced pluripotent stem cells;
ISC	iron-sulfur clusters;
ISCU	iron-sulfur cluster assembly enzyme;
KEAP1	Kelch-like ECH-associated protein 1;
KIKO	knock-in/knock-out;
MMP	mitochondrial membrane potential;
mRNA	messenger ribonucleic acid;
mTFA	mitochondrial transcription factor A;
mTOR	mammalian target of rapamycin;
NDUFS3	NADH: ubiquinone oxidoreductase core subunit s3;
NE	not evaluated;
NF-kB	nuclear factor kappa-light-chain-enhancer of activated B cells;
NQO1	NAD(P)H quinone oxidoreductase 1;
NRF1	nuclear respiratory factor 1;
NRF2	nuclear factor erythroid 2–related factor 2;
NSCs	neural stem cells;
OGDH	8-oxoglutarate dehydrogenase E1 component;
PDH	pyruvate dehydrogenase;
PGC-1α	peroxisome proliferator-activated receptor-γ (PPARγ) coactivator 1 alpha;
ROS	reactive oxygen species;
RSL-3	RAS-selective lethal 3;
SDH-A	succinate dehydrogenase complex, subunit A;
SOD	superoxide dismutase;
TERT	telomerase reverse transcriptase;
4-HNE	4-hydroxynonenal;
8-oxodG	8-oxo-2′-deoxyguanosine
